# Elimination of 4T1 Mammary Tumor Cells by BALB/cBy UBC-GFP Transgenics following Stable Inheritance of the H-2^b^ MHC Allele

**DOI:** 10.4049/immunohorizons.2200101

**Published:** 2023-01-13

**Authors:** Candice A. Grzelak, Cyrus M. Ghajar

**Affiliations:** * Public Health Sciences Division/Translational Research Program, Fred Hutchinson Cancer Center, Seattle, WA; and; † Human Biology Division, Fred Hutchinson Cancer Center, Seattle, WA

## Abstract

The human ubiquitin C promoter (UBC)–driven GFP-transgenic mouse (UBC-GFP) transgene integration site was mapped recently to chromosome 17, linked closely to the MHC locus. In this study, we demonstrate a functional consequence of this insertion site in the backcrossed UBC-GFP BALB/c congenic strain [CByJ.B6-Tg(UBC-GFP) 30Scha/J]: rejection of transplanted “syngeneic” 4T1 mammary tumor cells. Rejection of BALB/c-derived 4T1 cells is in all likelihood a consequence of MHC mismatch due to stable inheritance of C57BL/6-derived H-2^b^ (rather than prototypical H-2^d^) by the BALB/c UBC-GFP strain. These data are a valuable resource to researchers who have previously employed the UBC-GFP congenic strain for attempted syngeneic MHC-matched and allogenic MHC-mismatched studies, as their data likely require reinterpretation. Further, this study reemphasizes the impact of mapping transgene integration sites of commonly used mouse strains as a way of increasing scientific rigor and reproducibility.

## Introduction

For years, tumor progression and metastasis have been modeled in immune-compromised animals (1). With the clinical acceleration of immunotherapies and the need to study complex mechanisms of resistance, this is no longer justifiable (2). Transplantation of tumor cells into syngeneic, immune-competent hosts has been suggested as a more relevant experimental model. In this study, to measure progression and therapeutic response, tumor cells are frequently infected with virus encoding bioluminescent and/or fluorescent proteins prior to transplantation. The issue is that these proteins, which are derived from other species, generate an adaptive immune response when transplanted into immune-competent hosts (3–8). The potential implications are profound.

Our group (4) recently highlighted that fluorescent protein immunogenicity is so profound in BALB/c mice that it attenuates progression of primary (orthotopic/mammary gland inoculated) tumors and almost totally blunts metastasis of the highly metastatic mammary cancer cell line 4T1 (9). This coincided with the presence of CD8^+^ T cells directed against the dominant antigenic epitope of enhanced GFP (4). To solve this potent experimental artifact, we reasoned that transgenics tolerized centrally to GFP [i.e., transgenics that express GFP in a significant proportion of thymic APCs (6)] would eliminate the CD8^+^ T cell response against xenoantigen eGFP. Indeed, use of a GFP-tolerized strain (e.g., *Cx3cr1-GFP*, BALB/c mice) completely eliminated the CD8^+^ T cell–driven immune response against GFP, allowed unabated primary tumor progression of GFP-expressing 4T1 mammary tumor cells, and permitted outgrowth of GFP^+^ metastasis within this immune-competent setting (4). We have observed similar data when employing the C57BL/6 centrally tolerized strain, *Aire-GFP* (6, 10) (C.A. Grzelak and C.M. Ghajar, unpublished observations).

Prior to publication of this work, we wished to conduct parallel experiments in an additional GFP-tolerized strain for the sake of rigor. We initially selected ubiquitin C promoter (UBC)-GFP mice for this purpose because Malhotra et al. (6) established this strain [C57BL/6-Tg(UBC-GFP)30Scha/J; 004353; The Jackson Laboratory] (11) as the most robustly tolerized to GFP out of 14 C57BL/6 transgenic mouse strains expressing GFP, YFP, or CFP in one or more tissue compartments. Because we wished to test a BALB/c-derived tumor cell line, we opted for the congenic UBC-GFP backcross onto BALB/cBy available from The Jackson Laboratory [CByJ.B6-Tg(UBC-GFP) 30Scha/J; 007076]. We hypothesized that inoculating UBC-GFP hosts with GFP-4T1 cells would mirror results obtained in *Cx3cr1-GFP* transgenics.

To our surprise, transplantation of GFP-expressing 4T1 cells into BALB/cBy UBC-GFP mice resulted in more extensive tumor rejection than that seen with the BALB/cBy immune-competent control. But unlike 4T1 tumors in wild-type (WT) hosts that frequently lose GFP expression (4), these tumors retained GFP, adding to our confusion. Shortly thereafter, Liu et al. (12) published data mapping the chromosomal insertion site of the UBC-GFP transgene to the MHC locus. Following publication of their study, we were able to reconcile that our results were likely a consequence of MHC mismatch, thereby corroborating and extending the findings of Liu et al. (12) to demonstrate the functional outcome of transplanting syngeneic tumors into the UBC-GFP BALB/c model. These data are highlighted below.

This study emphasizes the importance of mapping transgene integration sites to enhance scientific rigor and reproducibility. It also provides additional evidence of the need to execute caution in the interpretation of data from syngeneic cell transplantation studies carried out using the CByJ.B6-Tg(UBC-GFP) 30Scha/J strain.

## Materials and Methods

### Institutional Animal Care and Use Committee and mouse strains

All mouse work was performed in accordance with Institutional Animal Care and Use Committee and American Association for Laboratory Animal Science guidelines under Fred Hutchinson Cancer Center–approved protocol 51075. Mouse strains used were purchased directly from The Jackson Laboratory and include: CByJ.B6-Tg(UBC-GFP)30Scha/J (007076), referred to as UBC-GFP; BALB/cByJ (001026); and NOD.CB17-Prkdcscid/J (001303), referred to as NOD-SCID.

### Cell culture and reagents

The 4T1 mammary tumor line was purchased from the Karmanos Cancer Institute. Generation of the 4T1-R26-FerH-eGFP cell clone is described below. Cells were propagated in high-glucose DMEM (11965-118; Thermo Fisher Scientific) containing 10% (v/v) FBS (Thermo Fisher Scientific) and 1% (v/v) penicillin/streptomycin antibiotic (15140122; Thermo Fisher Scientific) and cultured under standard conditions (5% CO_2_, 37°C).

### Generating the 4T1-R26-FerH-eGFP line

A custom *ROSA26* safe harbor donor cloning vector (containing ROSA26 left arm, custom MCS; and bGH polyA, ROSA26 right arm) was designed and constructed by GeneCopoeia (CS-SH250-01; available on request). EcoRI-HF and BamHI-HF were used to linearize the *ROSA26* donor vector. Gibson assembly was used to clone the “FerH promoter-ffLUC-eGFP” fragment from pFUGW-FerH-ffLuc2-eGFP (13) into the *ROSA26* donor vector within the multiple cloning site. Primer sequences used for Gibson cloning were: forward, 5′-CGTCTCGAGCTCAAGCTTCGCGGCCTGAAATAACCTCTGAA-3′; and reverse, 5′-AACTAGAAGGCACAGGGATCCCTACTTGTACAGCTCGTCCA-3′.

Insertion of FerH-ffLUC-eGFP into the donor vector was verified via Sanger sequencing, generating “Rosa26 donor_FerH-ffLUC-eGFP” (9,623 bp). To achieve expression of GFP only, Rosa26 donor_FerH-ffLUC-eGFP underwent double restriction digestion with MluI (2,158 bp) and AgeI (5,287 bp) to remove the “FerH-ffLUC” fragment. Gibson assembly was used to reinsert the ferritin H chain (“FerH”) promoter fragment (1,268 bp), 3′ of the *ROSA26* left homology arm into the remaining 6,494-bp vector backbone. Sequencing of Rosa26 donor_FerH-eGFP confirmed the final vector sequence (7,679 bp).

The 4T1 mammary tumor cells (100,000 cells) were transfected at 50% confluence using Xfect Transfection Reagent (Clontech) per the manufacturer’s instruction. The Rosa26 donor_FerH-eGFP (15 µg) and left and right TALEN constructs (2.5 µg each) were transfected with Xfect (6 µl/well). Left and right *ROSA26* TALEN constructs were purchased from GeneCopoeia (Genome-TALER mouse ROSA26 safe harbor gene knock-in kit, without donor; SH075). Once GFP expression diminished from the donor-alone control well (∼9 d following transfection), 4T1 cells cotransfected with TALENs plus donor were sorted to enrich for GFP^+^ cells.

After GFP enrichment/expansion, single-cell clones were generated by plating GFP^+^ cells into 96-well plates by limiting dilution (0.3 cell/well). Colonies were detectable 11 d after plating. At this time point, genomic DNA (gDNA) was extracted (QuickExtract; Lucigen) and samples screened by PCR. To screen for transgene knock-in to the *ROSA26* locus, a forward primer was designed to bind within the FerH-eGFP transgene (R26_right arm_fwd) and reverse primer designed to bind within the endogenous *ROSA26* locus (R26_right arm_rev). Primer sequences were R26_right arm_fwd, 5′-GCGGTGGGCTCTATGGTGTAC-3′; and R26_right arm_rev, 5′-ACATCCACCTGGAAACCATTAATGG-3′, which generated a 976-bp product. To screen 4T1-R26-FerH-GFP single-cell clones, PCR reactions contained 1× Phusion HF buffer, 200 μM deoxynucleoside triphosphate, 0.5 μM forward primer, 0.5 μM reverse primer, 0.02 U/μl Phusion Hot Start II, and 1 μl DNA template (QuickExtract). PCR conditions were: initial denature (98°C, 45 s); denature (98°C, 10 s), annealing (65°C, 30 s), and extension (72°C, 2 min) for 34 cycles; final extension (72°C, 10 min); and hold at 4°C. gDNA extracted from 4T1 WT cells (negative control), cells plus *ROSA26* donor alone (negative control), bulk unsorted cells plus *ROSA26* donor plus TALENs (negative control), and bulk sorted cells plus *ROSA26* donor plus TALENs (positive control) were included for each line. Following PCR amplification, samples were run on a 1% agarose/TAE gel.

Clones positive for insertion at the *ROSA26* locus by PCR were expanded out from 96-well plates into T75s and frozen back. A second round of PCR was performed on expanded clones to confirm insertion of the expanded population a second time. A single-cell clone was ultimately chosen based off the: 1) ability to maintain homogenous GFP expression in culture; 2) ability to grow in culture; and 3) morphology most closely resembling the parental 4T1 tumor line. This led to choosing the following clone, “4T1-R26-FerH-GFP A12,” subsequently known as “4T1-R26-FerH-GFP.”

### Orthotopic mammary transplantation model

4T1 tumor cells expressing FerH-driven eGFP and integrated at the *ROSA26* locus (4T1-R26-FerH-eGFP) were injected orthotopically (750,000 cells/mouse) into the fourth mammary fat pad of 6- to 7-wk-old female NOD-SCID (immune-compromised; *n* = 6), BALB/cBy (immune-competent; *n* = 9), or UBC-GFP (GFP-tolerized, BALB/cBy congenic strain; *n* = 9) mice in a 50-µl solution of 1:1 LrECM (growth factor–reduced Cultrex; Trevigen)/PBS. Mice were euthanized 3 wk following orthotopic injection, with the primary tumor intact. Following euthanasia, mice were perfused with 3 ml 1× PBS via the inferior vena cava with simultaneous ligation of the portal vein (to directly target the liver). This was followed by 15 ml 1× PBS delivered by intracardiac perfusion via the left ventricle to target the remaining organs. Primary tumors were collected from all three groups; spleens were collected from immune-competent and UBC-GFP mice; and tails were collected from the UBC-GFP group given the unexpected rejection of the 4T1-R26-FerH-eGFP tumor line.

### Mammary tumor measurements

Calipers were used to measure the long (L) and short (w) axes of the tumor. Volume was calculated using the equation V = 0.5 Lw^2^. Tumor measurements were recorded starting day 4 post–tumor cell injection three times per week.

### Tumor immunofluorescence

#### Sectioning

Following perfusion with 1× PBS, primary tumors were dissected. Half of the tumor was flash frozen in OCT using liquid nitrogen. Cryosections (12 µm) of tumor were cut using a Leica Cryostat CM3050 S (Leica Microsystems) and placed onto Superfrost Plus glass slides. After sections had dried, slides were placed back to back, wrapped in Kimwipes, wrapped in foil, and stored unfixed at −80°C until required.

#### Immunofluorescence

Wrapped slides were placed at 37°C for 10 min to allow any condensation to evaporate. Sections were placed into a Coplin jar and then immediately fixed with 10% neutral buffered formalin (HT501128-4L; Sigma-Aldrich) for 20 min at room temperature (RT). Following washing with copious 1× PBS with agitation (3 × 5 min), sections were permeabilized using 0.5% (v/v) Triton X-100/PBS for 20 min at RT. Slides were washed again and then blocked using 10% (v/v) donkey serum plus Li-COR Odyssey blocking buffer (927-40000; Li-COR Biosciences) for 1 h at RT. The following primary Abs were then added to the slides (overnight at 4°C), diluted in Odyssey blocking buffer at the following concentrations: cytokeratin 18 (CK18; 1:100; GP11; Progen) and GFP (1:1,000; ab13970; Abcam). The following day, slides were washed (three times for 5 min each), and the appropriate highly cross-adsorbed secondary Abs were added at 1:400, diluted in Odyssey blocking buffer: donkey anti-chicken CF488A (20166; Biotium) and donkey anti–guinea pig CF568 (20377; Biotium). Following incubation in secondary Ab for 45 min at RT, slides were washed for a final time in PBS (three times for 5 min each), and DNA was stained with Hoechst (1:10,000 in PBS) for 5 min at RT to visualize nuclei. Following another PBS wash, slides were mounted using Fluoromount-G (0100-01; Thermo Fisher Scientific), sealed using nail polish, and stored at 4°C prior to imaging.

#### Imaging

An entire cross section of each primary tumor was obtained (2.5×/0.12 M27 objective lens, 6 × 6 tile scan, 1,024 × 1,024 frame, speed 8) using 405, 488, and 555 laser lines on a Zeiss LSM700 confocal microscope. Hoechst (405), GFP (488), and CK18 (555) signal were captured following excitation with the listed laser. Insets were obtained using a 20×/0.55 numerical aperture air objective lens. Identical imaging acquisition settings were applied to all samples within the same study.

### UBC-GFP transgene detection

gDNA was extracted from mouse tails from UBC-GFP (on-study) and BALB/c mice (off-study, as a negative control) using the DNeasy Blood and Tissue Extraction Kit (69504; Qiagen) according to the manufacturer’s protocol. To screen for the UBC-GFP transgene, we used a PCR assay developed previously to map UBC-GFP transgene integration in the CByJ.B6-Tg(UBC-GFP)30Scha/J backcrossed strain closely to the MHC locus (12). Detection of this PCR product indicates stable inheritance of the UBC-GFP transgene at this site and as a consequence inheritance of H-2^b^. The forward primer (H-2_FOR; 5′-CACACACACACACGTCCTTG-3′) resides within an endogenous flanking sequence on chromosome 17 (Chr 17), whereas the reverse primer (UBC_REV; 5′-TCCATTCAAGACTCGGGAAC-3′) resides within the UBC-GFP transgene itself. H-2_FOR and UBC_REV will generate a 1,059-bp product if integration is retained at this site. PCR reactions contained 1× Phusion HF buffer, 200 μM deoxynucleoside triphosphate, 0.5 μM forward primer, 0.5 μM reverse primer, 0.02 U/μl Phusion Hot Start II, and gDNA template (80 ng). PCR conditions were: initial denature (98°C, 10 s); denature (98°C, 1 s), annealing (62°C, 5 s), and extension (72°C, 20 s) for 33 cycles; final extension (72°C, 5 min); and hold at 4°C. Following PCR amplification, samples (50 μl) were run on a 1% (w/v) agarose/1× TAE gel containing 1 μg/ml ethidium bromide (1610433; Bio-Rad Laboratories) at 100 V for 40 min. A 1-kb DNA ladder (GeneRuler, SM0313; Thermo Fisher Scientific) (10 μl/lane) was used to assess product size.

### Immunospot assay

Following mouse harvest, spleens were placed into 5 ml of 1× CTL-Wash media plus 1% (v/v) penicillin/streptomycin (#CTLW-010; Cellular Technology Limited) on ice in a 6-cm dish until ready to process. To ensure high viability of samples, the time spleens were left on ice between harvesting, and processing was kept to a minimum. Spleens were processed according to the manufacturer’s instructions (Cellular Technology Limited) to cryopreserve using the CTL-Cryo ABC Media Kit (CTLC-ABC-100; Cellular Technology Limited). Splenocyte samples were frozen back at 2.0 × 10^7^/vial and stored in the liquid N_2_ until all samples were collected.

Mouse IFN-γ single-color fluorospot assays (mT1000Fp and mT01; Cellular Technology Limited) were purchased precoated from Cellular Technology Limited and carried out according to the manufacturer’s protocol. Specifically, following sample cryorevival and washing in 1× CTL anti-aggregate wash medium (CTL-AA-005; Cellular Technology Limited), viable splenocyte samples (400,000 cells/well) were plated in CTL-Test medium (#CTLT-005; Cellular Technology Limited) in duplicate with the following: 1) Con A (1 μg/ml; C0412-5MG; Sigma-Aldrich); 2) eGFP_(200-208)_ peptide (HYLSTQSAL; 1 μg/ml; Elim Biopharmaceuticals); or 3) FLU_(147-155)_ peptide (TYQRTRALV; 1 μg/ml; Elim Biopharmaceuticals). Con A (positive control) is an Ag-independent mitogen, stimulating T cell activation. eGFP (HYLSTQSAL) is the major antigenic epitope to eGFP, presented by H2-K^d^, which is typically inherited by BALB/c strains (3). FLU (TYQRTRALV) is a peptide to influenza nucleoprotein presented by H2-K^d^, used as a negative control peptide (14). Following culture at 37°C with Con A, eGFP, or FLU peptide for 24 h, IFN-γ detection and development was carried out according to the manufacturer’s protocol. After the plate was dry, IFNγ-CTLRed fluorescent spots were visualized and counted using an ImmunoSpot S6 Analyzer (Cellular Technology Limited) with ImmunoSpot software. Fluorescent signal was captured using the 690 filter and SmartCount applied to determine the number of red fluorescent IFN-γ spots per well. The average number of spots were determined per well and plotted in Prism. Samples were excluded if the Con A control did not respond appropriately.

## Results

**FIGURE 1.**
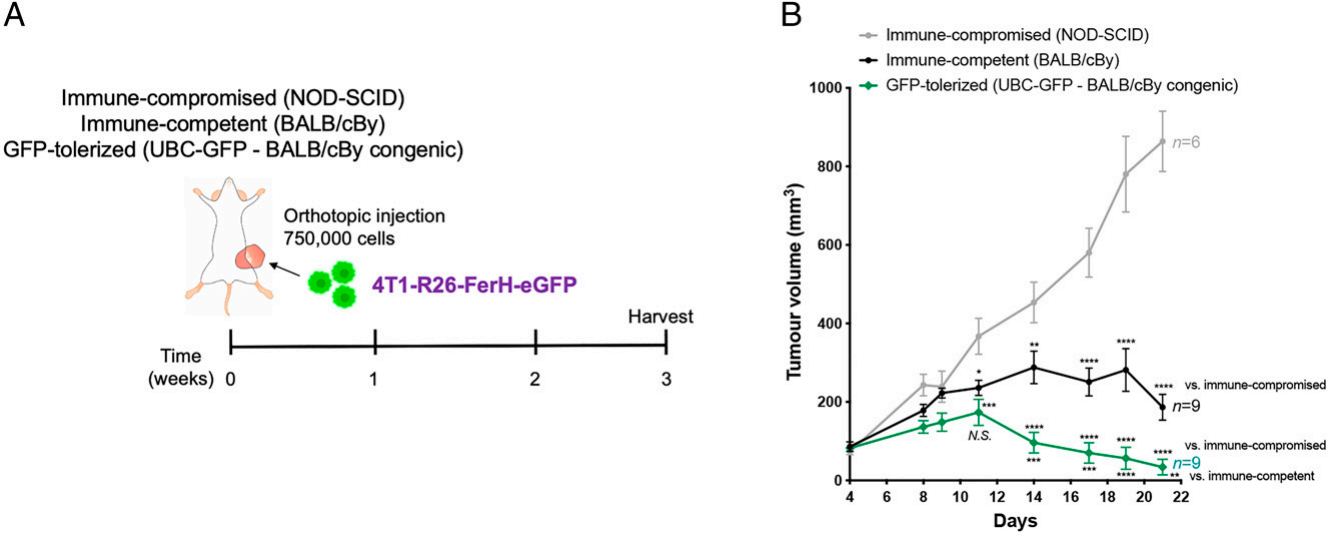
Tumor rejection of GFP-expressing 4T1 mammary tumor cells injected orthotopically into BALB/c UBC-GFP mice. (**A**) Experimental schematic. To determine whether GFP-tolerized mice permit primary tumor take and metastasis progression in an immune-competent setting, immune-compromised (NOD-SCID; positive control), immune-competent (BALB/cBy; negative control), and the BALB/c GFP-tolerized mouse strain UBC-GFP (007076; The Jackson Laboratory) were injected with 750,000 GFP-expressing 4T1 tumor cells in the fourth mammary fat pad. 4T1-R26-FerH-eGFP is a BALB/c-derived mammary tumor line that was engineered to express GFP integrated specifically at the *ROSA26* locus, with GFP expression driven by the introduced *FerH* promoter. (**B**) Tumor volume measurements were obtained between days 4 and 21 post–mammary fat pad injection in the three mouse strains: immune-compromised (NOD-SCID; gray), immune-competent (BALB/c; black), or BALB/c UBC-GFP (green). Mean ± SEM (*n* = 6–9/group, as indicated on graph). Two-way ANOVA with Tukey multiple-comparisons test. *p = 0.05, **p = 0.01, ***p = 0.001, ****p < 0.0001.

We wished to determine whether the use of an immune-competent host tolerized centrally to GFP would enable unadulterated modeling of primary tumor and metastatic progression (that is, modeling uninfluenced by the host immune response to foreign Ags like GFP) when mice are implanted with tumor cells expressing GFP. To do so, we orthotopically transplanted UBC-GFP; BALB/cBy congenics with 4T1-GFP cells using immune-compromised (NOD-SCID) and immune-competent (BALB/cBy) mice as positive and negative controls, respectively (Fig. 1A). Tumor cells (750,000 cells/mouse) were delivered directly into the fourth mammary fat pad. We chose to inject a highly metastatic mammary tumor line [4T1 (9)] expressing GFP integrated specifically within the *ROSA26* locus and driven by the FerH promoter (4T1-R26-FerH-eGFP).

**FIGURE 2.**
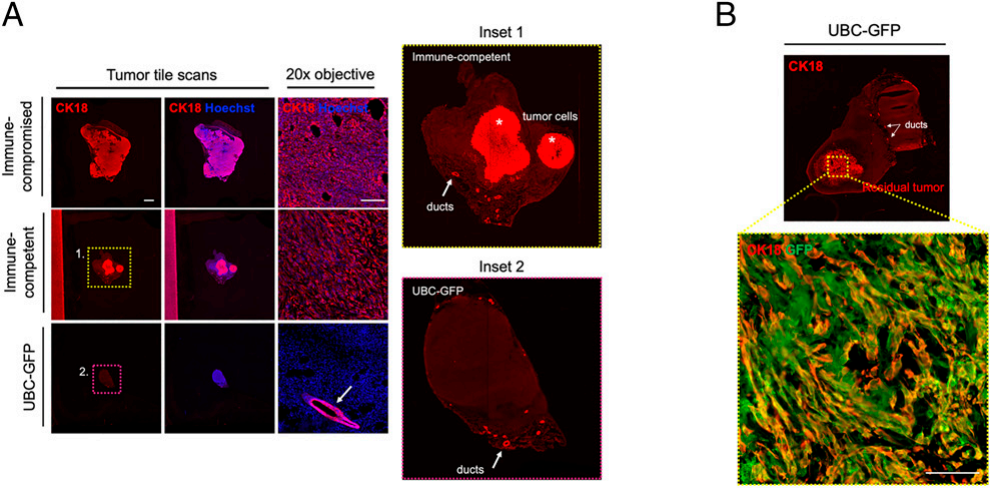
Immunofluorescence of UBC-GFP tumor remnants confirms outright rejection of 4T1 tumor cells and demonstrates residual foci maintain GFP expression. Immunofluorescence for CK18 (red; both a tumor cell and mammary duct marker) and Hoechst (blue) for nuclei in primary tumor sections taken from immune-compromised (NOD-SCID), immune-competent (BALB/c), or UBC-GFP (BALB/c) mice. (**A**) Representative tumor cross sections (2.5× objective, 6 × 6 tile scan) from primary tumors taken from the three mouse groups. CK18^+^ staining either depicts tumor cells (*) or intact mammary ducts (arrow) (e.g., inset 1, immune-competent). CK18^+^ tumor foci were observed in the mammary fat pad of 6 of 6 (100%) immune-competent BALB/c mice. Alternatively, mice that have completely rejected the primary tumor only display ductal expression (e.g., inset 2, UBC-GFP). CK18^+^ tumor foci were observed in only 2 of 6 (33%) UBC-GFP mice. Scale bars: tile scans, 2,000 μm; 20× objective, 100 μm. (**B**) Both residual tumors containing CK18^+^ tumor foci in UBC-GFP mice still expressed GFP (green) within CK18^+^ tumor cells. Scale bar, 100 um.

**FIGURE 3.**
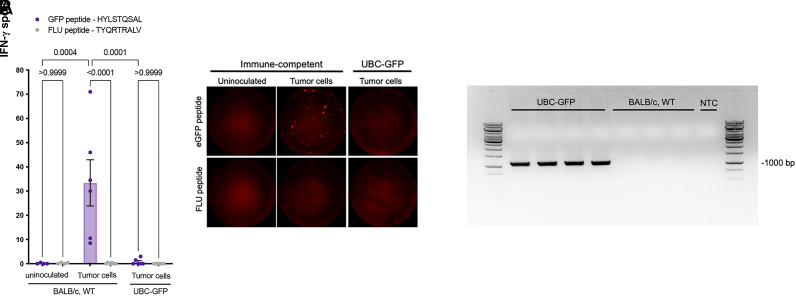
Inheritance of the C57BL/6-associated H2-K^b^ allele in BALB/c UBC-GFP mice is responsible for GFP-expressing 4T1 tumor cell rejection following MHC mismatch. (**A**) Immunospot assay confirms the absence of a H2-K^d^–restricted anti-GFP CTL immune response to inoculation with 4T1-R26-FerH-eGFP tumor cells in UBC-GFP mice only. Splenocytes were isolated from uninoculated BALB/c or 4T1-R26-FerH-eGFP inoculated mice (BALB/c or UBC-GFP) at day 21 post-injection. Cryopreserved splenocytes (400,000/well) were seeded, and following incubation either with H-2K^d^ eGFP peptide (HYLSTQSAL) or FLU peptide (TYQRTRALV) as a negative control for 24 h, the number of IFN-γ spots (red) were determined. This indicates the number of functional H2-K^d^–restricted GFP-specific CTLs per well. Mean ± SEM; one-way ANOVA with Tukey multiple-comparisons test. Representative wells are shown. (**B**) Confirming H2-K^b^ inheritance in UBC-GFP mice. PCR was used to confirm the UBC-GFP transgene integration site maps to the MHC locus (12). gDNA was extracted from tail snips (*n* = 4) taken from UBC-GFP or immune-competent BALB/c mice (*n* = 4) as a negative control for the assay. PCR was subsequently used to amplify the UBC-GFP transgene. Forward primer (H-2F) is located within the 5′ chromosomal flanking sequence to the UBC-GFP transgene, with the reverse primer (UBC R) located within the transgene itself. Amplification of a 1,059-bp product confirms UBC-GFP mice in this study maintain integration of the transgene at the locus identified by Liu et al. (12), which is on Chr 17 and closely linked to the MHC locus. NTC, no template control.

To our surprise, tumors were rejected in the GFP-tolerized UBC-GFP strain (Fig. 1B, green line). Dynamics of tumor growth were blunted significantly in UBC-GFP mice as compared with the immune-compromised group and even the immunocompetent controls (Fig. 1B, black line). CK18 marked both tumor cell foci (*) and remaining mammary ducts (white arrows) within the residual fat pad across strains (Fig. 2A). All remnant tumors (6 of 6; 100%) that we reviewed in immune-competent BALB/cBy mice had detectable CK18^+^ tumor cell foci (Fig. 2A, inset 1), whereas only 2 of 6 (33%) UBC-GFP mice contained any remaining tumor cells (*p* = 0.06, Fisher exact test, two-tailed). This indicated complete tumor rejection in the majority of the UBC-GFP cohort, which was contrary to our expectations given that 100% (9 of 9) of tumors took in another tolerized strain: *Cx3cr1-GFP;CCR2-RFP* mice, with final tumor burden similar to that of NOD-SCID mice (*p* = 0.78) (4). Interestingly, when we looked at the two UBC-GFP tumors with remaining tumor foci, these were still GFP^+^. This suggested that rejection of the tumor in this strain was not mediated by a GFP-specific CD8^+^ T cell response. Lack of a H2-K^d^–restricted anti-GFP CTL immune response in UBC-GFP mice following inoculation with 4T1-R26-FerH-eGFP tumor cells (Fig. 3A) corroborated these data and added to our confusion. Importantly, we documented that a GFP-directed response was intact and detectable by immunospot in WT BALB/cBy (Fig. 3A), suggesting that the anti-GFP CTL response was indeed absent or below the level of detection in UBC-GFP transgenics. What, then, was the cause of tumor rejection if it was not immunity raised against a potent fluorescent xenogen?

Recently, Liu et al. (12) published data mapping the chromosomal insertion site of the UBC-GFP transgene to the MHC locus. As a consequence, the CByJ.B6-Tg(UBC-GFP) 30Scha/J strain we had employed likely expressed H2-K^b^ inherited from the C57BL/6 genome, rather than H2-K^d^. This would explain the robust tumor rejection (due to MHC mismatch) following injection of BALB/c-derived 4T1-R26-FerH-eGFP cells. To confirm that our cohort of mice maintained integration at the site identified by Liu et al. (12), we extracted gDNA from UBC-GFP tails. PCR indicated maintained inheritance of the UBC-GFP transgene at this locus, following generation of the anticipated 1,059-bp product (Fig. 3B). This implies inheritance of H2-K^b^.

In light of these data, lack of a H2-K^d^–restricted anti-GFP CTL immune response in inoculated UBC-GFP mice does not necessarily reflect an absent response to GFP (Fig. 3A). To conclude, most importantly, we suspect rejection of inoculated syngeneic cells is due to MHC mismatch and caution further use of this strain for transplant studies.

## Discussion

In this study, we describe outright rejection of GFP-expressing mammary tumor cells implanted orthotopically in UBC-GFP mice, despite ubiquitous expression of GFP throughout the host. This was at complete odds with our initial hypothesis and data derived from other GFP-tolerized strains (4) (C.A. Grzelak and C.M. Ghajar, unpublished observations). Examination of residual tumors or remnant mammary fat pad by immunofluorescence confirmed complete rejection of GFP-4T1 cells in the majority (66%) of UBC-GFP mice. In mice with residual tumor foci, 4T1 cells retained GFP expression, which was puzzling initially. Ultimately, we confirm that the UBC-GFP transgene integration site in our cohort maps to Chr 17 and posit rejection of syngeneic 4T1 tumor cells results from coinheritance of the C57BL/6 H-2^b^ MHC locus with the UBC-GFP transgene in the commercially available backcrossed BALB/c transgenic. Thus, our data confirm this recently documented coinheritance (12) and highlight the functional consequence of transplanting syngeneic tumor cells into CByJ.B6-Tg(UBC-GFP) 30Scha/J hosts. These data should steer any investigator wishing to conduct syngeneic cellular or organ transplant studies away from this transgenic.

Of note, before employing use of this strain, we had engaged all available paths to ensure that CByJ.B6-Tg(UBC-GFP) 30Scha/J hosts were suitable for our studies. We inquired directly with The Jackson Laboratory as to what specific genetic information they had on this congenic cross and specified that we wished to do a syngeneic tumor transplantation study. We were informed that the CByJ.B6-Tg(UBC-GFP) 30Scha/J strain had been screened using a 48–single nucleotide polymorphism (SNP) panel. One of the markers on Chr 17 was segregating, but where this SNP was located was not disclosed. After moving forward with our study and sharing our data, The Jackson Laboratory revealed the specific location of the SNP on Chr 17; it was located within MHC class II. Comprehensive mapping of the UBC-GFP insertion site by Liu et al. (12) provided independent confirmation of MHC coinheritance and allowed us to indisputably verify that the mice in our UBC-GFP cohort had inherited the UBC-GFP transgene within the H-2^b^ locus.

Our study highlights the need to rigorously conduct transgene integration site mapping in transgenic mice and to disclose these results in a transparent fashion. Wider adaptation of such practices is a simple step toward improved scientific rigor and reproducibility, a topic that regularly garners attention (15–19). Our study also provides additional functional evidence that caution is required when interpreting results from former studies that employ the CByJ.B6-Tg(UBC-GFP) 30Scha/J strain in syngeneic transplantation settings.

## References

[1] Holen I., V. Speirs, B. Morrissey, K. Blyth. 2017. In vivo models in breast cancer research: progress, challenges and future directions. Dis. Model. Mech. 10: 359–371. 28381598 10.1242/dmm.028274 PMC5399571

[2] Binnewies M., E. W. Roberts, K. Kersten, V. Chan, D. F. Fearon, M. Merad, L. M. Coussens, D. I. Gabrilovich, S. Ostrand-Rosenberg, C. C. Hedrick, et al 2018. Understanding the tumor immune microenvironment (TIME) for effective therapy. Nat. Med. 24: 541–550. 29686425 10.1038/s41591-018-0014-x PMC5998822

[3] Gambotto A., G. Dworacki, V. Cicinnati, T. Kenniston, J. Steitz, T. Tüting, P. D. Robbins, A. B. DeLeo. 2000. Immunogenicity of enhanced green fluorescent protein (EGFP) in BALB/c mice: identification of an H2-Kd-restricted CTL epitope. Gene Ther. 7: 2036–2040. 11175316 10.1038/sj.gt.3301335

[4] Grzelak C. A., E. T. Goddard, E. E. Lederer, K. Rajaram, J. Dai, R. E. Shor, A. R. Lim, J. Kim, S. Beronja, A. P. W. Funnell, C. M. Ghajar. 2022. Elimination of fluorescent protein immunogenicity permits modeling of metastasis in immune-competent settings. Cancer Cell 40: 1–2. 34861158 10.1016/j.ccell.2021.11.004 PMC9668376

[5] Han W. G., W. W. Unger, M. H. Wauben. 2008. Identification of the immunodominant CTL epitope of EGFP in C57BL/6 mice. Gene Ther. 15: 700–701. 18288211 10.1038/sj.gt.3303104

[6] Malhotra D., J. L. Linehan, T. Dileepan, Y. J. Lee, W. E. Purtha, J. V. Lu, R. W. Nelson, B. T. Fife, H. T. Orr, M. S. Anderson, et al 2016. Tolerance is established in polyclonal CD4(+) T cells by distinct mechanisms, according to self-peptide expression patterns. Nat. Immunol. 17: 187–195. 26726812 10.1038/ni.3327 PMC4718891

[7] Morris J. C., M. Conerly, B. Thomasson, J. Storek, S. R. Riddell, H. P. Kiem. 2004. Induction of cytotoxic T-lymphocyte responses to enhanced green and yellow fluorescent proteins after myeloablative conditioning. Blood 103: 492–499. 14512305 10.1182/blood-2003-07-2324

[8] Stripecke R., M. Carmen Villacres, D. Skelton, N. Satake, S. Halene, D. Kohn. 1999. Immune response to green fluorescent protein: implications for gene therapy. Gene Ther. 6: 1305–1312. 10455440 10.1038/sj.gt.3300951

[9] Miller F. R., B. E. Miller, G. H. Heppner. 1983. Characterization of metastatic heterogeneity among subpopulations of a single mouse mammary tumor: heterogeneity in phenotypic stability. Invasion Metastasis 3: 22–31. 6677618

[10] Gardner J. M., J. J. Devoss, R. S. Friedman, D. J. Wong, Y. X. Tan, X. Zhou, K. P. Johannes, M. A. Su, H. Y. Chang, M. F. Krummel, M. S. Anderson. 2008. Deletional tolerance mediated by extrathymic Aire-expressing cells. Science 321: 843–847. 18687966 10.1126/science.1159407 PMC2532844

[11] Schaefer B. C., M. L. Schaefer, J. W. Kappler, P. Marrack, R. M. Kedl. 2001. Observation of antigen-dependent CD8+ T-cell/ dendritic cell interactions in vivo. Cell. Immunol. 214: 110–122. 12088410 10.1006/cimm.2001.1895

[12] Liu S., J. R. Lockhart, S. Fontenard, M. Berlett, T. M. Ryan. 2020. Mapping the chromosomal insertion site of the GFP transgene of UBC-GFP mice to the MHC locus. J. Immunol. 204: 1982–1987. 32122998 10.4049/jimmunol.1901338

[13] Day C. P., J. Carter, C. Bonomi, D. Esposito, B. Crise, B. Ortiz-Conde, M. Hollingshead, G. Merlino. 2009. Lentivirus-mediated bifunctional cell labeling for in vivo melanoma study. Pigment Cell Melanoma Res. 22: 283–295. 19175523 10.1111/j.1755-148X.2009.00545.x PMC2726997

[14] Rötzschke O., K. Falk, K. Deres, H. Schild, M. Norda, J. Metzger, G. Jung, H. G. Rammensee. 1990. Isolation and analysis of naturally processed viral peptides as recognized by cytotoxic T cells. Nature 348: 252–254. 1700304 10.1038/348252a0

[15] Day C.-P., E. Pérez-Guijarro, A. Lopès, R. S. Goldszmid, M. Murgai, L. Wakefield, G. Merlino. 2022. Recognition of observer effect is required for rigor and reproducibility of preclinical animal studies. Cancer Cell 40: 231–232. 35180384 10.1016/j.ccell.2022.01.015

[16] National Institutes of Health. 2020. ACD Working Group on Enhancing Rigor, Transparency, and Translatability in Animal Research. Available at: https://acd.od.nih.gov/working-groups/eprar.html. Accessed: October 13, 2022.

[17] Hines W. C., Y. Su, I. Kuhn, K. Polyak, M. J. Bissell. 2014. Sorting out the FACS: a devil in the details. Cell Rep. 6: 779–781. 24630040 10.1016/j.celrep.2014.02.021

[18] Prager E. M., K. E. Chambers, J. L. Plotkin, D. L. McArthur, A. E. Bandrowski, N. Bansal, M. E. Martone, H. C. Bergstrom, A. Bespalov, C. Graf. 2019. Improving transparency and scientific rigor in academic publishing. J. Neurosci. Res. 97: 377–390. 30506706 10.1002/jnr.24340 PMC12990824

[19] Wilson J. L., C. M. Botham. 2021. Three questions to address rigour and reproducibility concerns in your grant proposal. Nature 596: 609–610.

